# Factors influencing UK residents’ preferences in how psychologists present themselves online: a conjoint analysis during the early months of the COVID-19 pandemic

**DOI:** 10.1186/s12913-022-08356-w

**Published:** 2022-07-28

**Authors:** Magnus Jørgensen, Guido Makransky

**Affiliations:** 1 Department of Psychology, University of Copenhagen, Øster Farimagsgade 2A, 1353 Copenhagen, Denmark; 2 Present address: Department of Health Promotion and Development, University of Bergen, Årstadsveien 17, 5009 Bergen, Norway

**Keywords:** Self-disclosure, Specialization, Help-seeking, Preferences, Private practitioner, Mental health care marketing, COVID-19 fear

## Abstract

**Background:**

The COVID-19 pandemic has led to a surge in mental health issues in the UK and worldwide, yet many UK residents have not received the help they have needed. Earlier research has indicated that accommodating client preferences leads to better therapeutic outcomes, however, little is known about preferences in how psychologists present themselves online and what might facilitate or slow help-seeking individuals’ decision about whom to seek help from. Based on literature suggesting personal branding as an effective way for clients to choose between psychologists, we sought to investigate UK residents’ preferences for specialization and self-disclosure in online presentations of psychologists based on level of fear of COVID-19 and diagnostic status.

**Methods:**

A sample of 187 UK residents were surveyed mid-April 2020 and analyzed using a rating-based conjoint analysis with a fractional factorial design consisting of 22 profiles. Each profile consisted of six attributes (Years of experience, area of expertise, gender, self-disclosure, training institution and facial trustworthiness). Analyses of variance (ANOVA) were used to explore preferences for area of expertise and self-disclosure depending on fear of COVID-19. An independent sample t-test was done to explore preference for self-disclosure among diagnosed residents.

**Results:**

The conjoint model yielded a good fit (Kendall’s tau = .90, *p* < .001). Relative importance scores (RI) were highest for years of experience (RI = 28.84) and area of expertise (RI = 22.78). Residents with a high fear of COVID-19 preferred psychologists specialized in anxiety disorders and fear (also fear of COVID-19) more than residents with a low fear of COVID-19 (MD = .92, 95% CI = [.198, 1.64], *p* = .013). Residents with a high fear of COVID-19 also preferred self-disclosing psychologists more than residents with a low fear of COVID-19 (MD = 1.05, 95% CI = [.184, 1.92], *p* = .013). Diagnostic status was not associated with preference for self-disclosure.

**Conclusions:**

Listing de facto specialization in psychologist profiles might facilitate prospective clients’ decision-making process. Self-disclosure appears to be important for some clients, but more research is warranted.

## Introduction

In the early months of the COVID-19 pandemic, the United Kingdom (UK) was particularly badly affected by COVID-19 with 260.916 confirmed cases and 36.875 deaths as of 25^th^ of May, 2020—making it Europe’s worst-hit country at the time [1]. Unsurprisingly, UK residents also reported higher levels of mental health issues than normal, but many individuals chose not to contact primary care out of fear of contracting COVID-19 and further burdening the National Health Services (NHS) [2]. In this respect, the shift towards digital mental health was important in improving access to mental health services, however, less is known about how online presentations of psychologists might have facilitated or reduced accessibility for UK residents experiencing high levels of fear of COVID-19 or those in risk of exacerbation of preexisting mental health disorders [3, 4]. This is important as pre-pandemic research points to accommodation of client preferences as an important factor in facilitating service uptake, creating positive treatment outcomes, and reducing dropouts [5–7].

The significance of client preferences is also reflected in UK residents’ right to—in most cases—choose their own mental health care provider through the NHS (which is more common than referrals through private insurance and more affordable than self-referrals to private practice) [8–10]. Yet, when it comes to specific preferences regarding psychologist attributes, earlier studies have focused mostly on preferences in therapy sessions and on relatively fixed attributes in profile presentations (e.g., race/ethnicity, age, professional identity, and training institution of mental health care professionals etc.) [11–13]. Thus, psychologists with clinical competence relevant for treatment of fear of COVID-19 were left with little practical advice on how to present themselves towards help-seeking clients during the early months of the pandemic. To address this gap and to better understand how prospective clients can find a relevant psychologist during times of crisis, we pursue the following research question in the present study: Can personal branding attributes in online presentations of psychologists facilitate decision-making for individuals experiencing high fear or who risk exacerbation of a preexisting mental health disorder during a time of crisis? To answer this question, the article is organized as follows: First, our literature review covers findings from earlier conjoint studies followed by a section on each of the two personal branding attributes (specialization and self-disclosure) selected for the present study. At the end of each of these sections, we present hypothesis 1 and 2 respectively. In the methods section, we describe our sample, instruments and how we designed the conjoint profiles. Next, in the results section, we present estimates from our conjoint analysis followed by ratings of each attribute and ending with results from ANOVAs and t-tests used to test hypothesis 1 and hypothesis 2. Lastly, in the discussion section, we compare our findings to earlier studies. Conclusions and implications are stated at the end of the article.

### Literature review

#### Earlier conjoint studies

Conjoint analysis is widely used in applied marketing research and is considered state of the art for preference measurement [14, 15]. Recent studies investigating preferences in choice of mental health professional have employed conjoint analysis, but most of these studies have been done on student populations [13, 16, 17]. Lee et al. [11] investigated preference for five attributes of mental health professionals (Gender, race/ethnicity, age, professional identity and training institution) in a sample of international Korean students in the US. They found significant preferences regarding race/ethnicity, age, professional identity, and training institution. Ip et al. [12] did another study among college students in Hong Kong which investigated preferences for five attributes (professional background, training institution, age, race/ethnicity, and gender) and reported similar findings to Lee et al. [11]. Finally, Kaya et al. [13] conducted a study with a sample of Turkish university students, which showed significant preferences for three out of five attributes: training institution, age, and professional background. In conclusion, previous conjoint studies have found standard practitioner information, such as training institution, age, professional background as well as race/ethnicity, to be important attributes in students’ preference for mental health professionals. However, most of these attributes are generic, hard to change and don’t showcase the personal and professional qualities of individual mental health professionals. Thus, there is little to gain for practitioners wanting to improve their self-presentation—whether on personal websites or in public directories. To our knowledge, there is also no conjoint study in this field that has included images in rating profiles, thus, limiting the external validity of current findings.

#### Specialization

Advocates of personal branding suggest psychologists should emphasize unique professional and personal characteristics that help differentiate them from colleagues while also building a connection with prospective clients [18]. In this regard, specialization and self-disclosure has been discussed as possible ways to achieve that [18]. Specialization is increasingly desired by clients in health care and has also been associated with an increase in marketability, salary, self-esteem and professional credibility of psychologists [19]. Moreover, de facto specialization (e.g., specializing in a niche etc.) show similar benefits to formal (de jure) specializations while still being permissible for several mental health conditions within the UK Code of Non-broadcast Advertising and Direct & Promotional Marketing [18, 20–22]. However, according to Stout, it is not uncommon for psychotherapists to list as many as 20 different specialties on their website, thus, challenging the British Psychological Society’s and the American Psychological Society’s guidelines on delineating limits of competence while also making it harder for distressed clients to figure out which therapist has actual competence with their specific issue [18, 21, 23]. Thus, in the present study, we formulated our first hypothesis as follows: UK residents with a high fear of COVID-19 prefer psychologists who list anxiety disorders and fear (also fear of COVID-19) as their area of expertise.

#### Self-disclosure

Self-disclosure has mostly been studied as a phenomenon in therapy sessions—and studies indicate that as much as 90% of psychologists have self-disclosed during therapy [24]. However, research on the effects of self-disclosure is mixed [24]. Still, a number of studies indicate clients prefer therapists who self-disclose a past history of mental illness [25, 26]. In fact, self-disclosure has been associated with increased expectation of therapeutic success, a stronger working relationship, and therapists being perceived as more likeable, warm, sincere, socially attractive, and empathetic [25–27]. However, little research has looked at preferences for self-disclosure based on client diagnostic status—though the general benefits of increased client-therapist similarity would suggest that individuals with a mental health disorder or issue would show greater preference for self-disclosure compared to the normal population [28, 29]. In the present study, we thus formulated our second hypothesis as: UK residents a with high fear of COVID-19 or a mental health diagnosis prefer psychologists who self-disclosure a past history of mild mental illness.

## Method

### Sample

A total of 187 UK residents (95 females and 92 males) were surveyed online from 11 to 13^th^ of April 2020 using self-selected sampling. All respondents consented to participate, and responses were anonymous. Age ranged from 18 to 76 years, with a mean age of 41.45 years (SD = 15.6). The ethnicity of the sample was predominantly white (80.2%), and 23% of the respondents had a least one mental diagnosis. Respondents in our sample came from diverse backgrounds in terms of region, educational attainment, and occupation. The total score average for fear of COVID-19 was 20.35 (SD = 7.13) while the average mean score was 2.91 (SD = 1.02).

### Instruments

This study used a survey which consisted of a demographic questionnaire, the Fear of COVID-19 Scale, a rating-based conjoint measurement of preferences in choice of psychologist and a rating scale for psychologist attributes. Images for the psychologist profiles in the rating based conjoint measure were selected from the Chicago Face Database.

### Demographics questionnaire

The survey contained a brief demographics questionnaire asking respondents their age, ethnicity, geographic region, educational level, occupation, and mental health diagnostic status.

### The Fear of COVID-19 scale

The Fear of COVID-19 Scale (FCV-19S) specifically measures fear of COVID-19 with seven items being rated on a 5-point Likert Scale ranging from strongly disagree to strongly agree [30]. The scale has good psychometric properties and has been validated in several countries [30–33]. The scores on the scale are interpreted as follows: A minimum score on any given item is 1 (strongly disagree) and a maximum score is 5 (strongly agree). Total scores range from 7 to 35 and are calculated by adding all item scores together [30]. Higher total scores indicate greater fear of COVID-19.

### The Chicago Face Database (CFD)

CFD is a database of faces developed by the University of Chicago [34]. CFD consists of 158 facial images [34]. The facial expressions are neutral and vary with two genders and four ethnicities [34]. A subset of faces includes four alternative facial expressions (Angry, Fear, Happy closed mouth, Happy open mouth). Furthermore, all faces have been rated on the following variables: Fearful/Afraid, Angry, Attractive, Baby-faced, Disgusted, Dominant, Feminine, Happy, Masculine, Prototypic, Sad, Surprised, Threat, Trustworthy, Unusual. All faces were rated on a Likert scale from 1 (Not at all) to 7 (Extremely) with respect to other people of the same race and gender [34].

The current study used a total of 22 faces from the database (including trustworthiness ratings of each). Happy (with open mouth) faces were selected to enhance external validity of the psychologist profiles. Faces were selected depending on gender, facial trustworthiness, and ethnicity. Only white faces were used to limit the number of attributes to six which is recommended in conjoint analysis [35]. The facial trustworthiness scores ranged from 3.1 to 4.2. The average score was 3.6 for females and 3.5 for males. Facial trustworthiness scores were divided into two categories: High facial trustworthiness (Females: *M* = 3.9, Males: *M* = 3.8, Both: *M* = 3.8) and low facial trustworthiness (Females: *M* = 3.2, Males: *M* = 3.1, Both: *M* = 3.2).

### Rating-based conjoint measurement of preferences in choice of psychologist

A rating-conjoint measurement of preferences in choice of psychologist was developed specifically for this study. The measure employed a rating-based conjoint design with six attributes: years of experience x area of expertise x gender x self-disclosure x training institution x facial trustworthiness. Levels of attributes were as follows: (a) years of experience (two levels: 5 years (± 1) and 15 years (± 1) of experience), (b) area of expertise (three levels: (1) general clinical experience (2) anxiety disorders and fear, and (3) anxiety disorders and fear (also fear of COVID-19), (c) gender (two levels: male, female), (d) self-disclosure (two levels: mild depression/anxiety and no disclosure), € training institution (two levels: high: the University of Oxford/the University of St. Andrews/the University of Bath/the University of Cambridge and low: the University of West London, Staffordshire University, the University of Kent and Edge Hill University) and (f) facial trustworthiness (two levels: high and low).

**Fig. 1:**
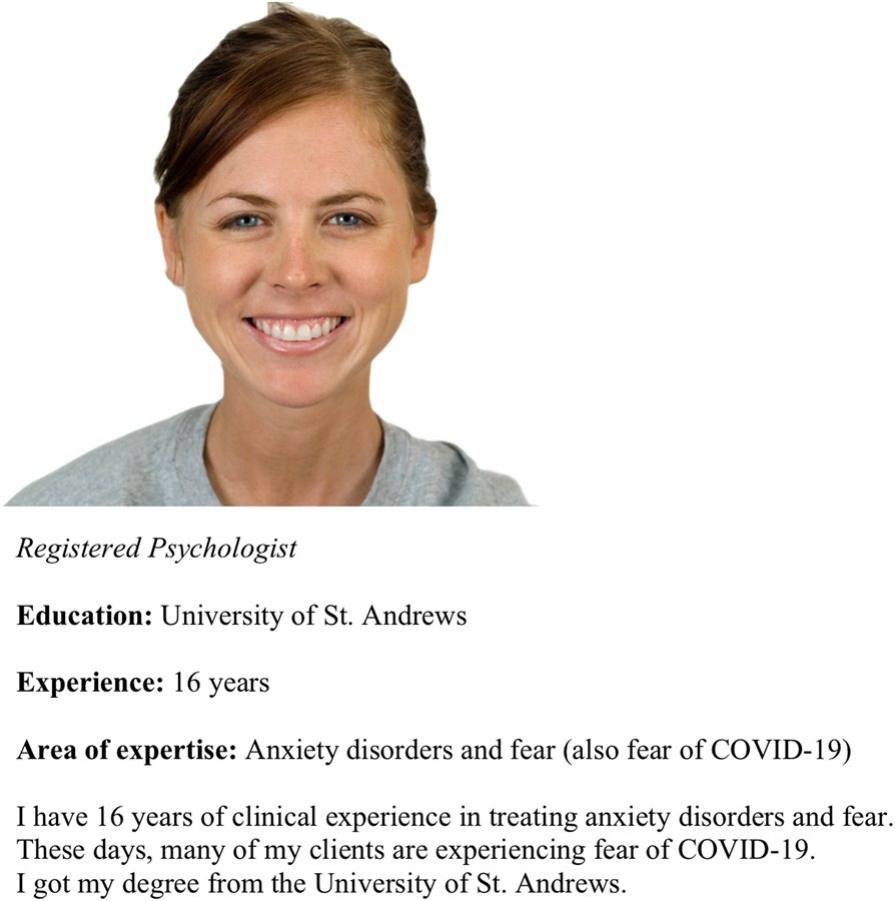
Example of a psychologist profile

In total, 22 psychologist profiles were rated on a Likert scale from 0 to 10 (not at all likely to highly likely), in terms of how likely respondents were to choose each psychologist based on their current mental health situation. Each profile contained a picture and short description (See Fig. 1). Profiles were displayed in a randomized sequence.

Figure 1 Example of a psychologist profile containing the following levels of each of the six attributes: years of experience (16 years), area of expertise (Anxiety disorders and fear (also fear of COVID-19), gender (female), self-disclosure (No disclosure), training institution (highly ranked university) and facial trustworthiness (Highly trustworthy face).

### Rating scale for psychologist attributes

Respondents were asked to rate six attributes on a Likert scale from 1 to 10. Attributes were similar, but not identical, to the attributes in the conjoint design. Attributes were phrased as the following statements: Psychologist is specialized in my problem, psychologist has personal experience with mental illness, psychologist has many years of experience, psychologist graduated from a highly ranked university, psychologist seems trustworthy, and psychologist is my preferred gender.

### Data analysis

The data was analyzed in SPSS with the Conjoint™ 8.0 procedure [36]. Initially, six attributes were created with two levels for each (expect area of expertise which had three levels). On this basis, SPSS generated an orthogonal plan for a fractional factorial design. The orthogonal plan consisted of 16 profiles to estimate attributes and utilities and six holdouts for cross-validation. Fractional factorial designs are used when the maximum number of combinations is deemed too high and respondent burden too heavy [15]. A full factorial design in this study would have included a total of 96 profiles.

## Results

### Relative importance scores (RI)

Relative importance scores for years of experience, area of expertise, gender, self-disclosure, training institution and facial trustworthiness are listed in Table 1. Kendall’s tau was applied to test the correlation between observed and estimated preferences and gave a correlation of 0.90 (*p* < 0.001) for psychologist profiles which indicates a good fit of the conjoint model on the observed preferences. To further check the validity of the utilities with a cross-validity test, Kendall’s Tau was computed for the six hold-out profiles and yielded a 0.73 correlation (*p* = 0.019), indicating a moderately strong relationship between observed and predicted rank orders for these profiles. In other words, the conjoint model seems to fit the observed preferences.

**Table 1:** Relative importance scores for attributes and utility estimates for attribute levels in rank order of preference

**Rank**	**Attribute level**	**Relative importance score**	**Utility Estimates**
	**Years of experience**	28.84	
1	15(± 1) years of experience		.543
2	5(± 1) years of experience		-.543
	**Area of expertise**	22.78	
1	Anxiety disorders and fear (also fear of COVID-19)		.262
2	Anxiety disorders and fear		-.158
3	General clinical experience		-.105
	**Gender**	14.68	
1	Female		.245
2	Male		-.245
	**Self-disclosure**	11.39	
1	Disclosure		.011
2	No disclosure		-.011
	**Training Institution**	11.11	
1	Higher ranked universities		.133
2	Lower ranked universities		-.133
	**Facial trustworthiness**	10.64	
1	More trustworthy faces		.070
2	Less trustworthy faces		-.070

To test whether the differences between attribute RI scores were significant, all possible 15 paired sample t-tests were conducted. In addition, The Holm-Bonferroni method was used to control for Type I errors. The paired sample t-tests revealed that years of experience, area of expertise, and gender were significantly higher than self-disclosure, training institution, and facial trustworthiness. Years of experience was the most important and area of expertise the second most important attribute. The paired sample t-test showed that gender was more important than self-disclosure, however, this was not significant after applying the Holm-Bonferroni method. Self-disclosure, training institution and facial trustworthiness did not differ significantly from one other.

### Utility Estimates

Utility estimates for years of experience, area of Expertise, gender, self-disclosure, training institution and facial trustworthiness are listed in Table 1 below.

### Rating of psychologist attributes

Respondents’ rating of the six attributes showed that general trust in the psychologist (M = 8.5, SD = 1.7), area of expertise (M = 8.2, SD = 1.7) and years of experience (M = 8.0, SD = 1.9) had the highest mean ratings. Self-disclosure (M = 6.9, SD = 2.4), training institution (M = 6.5, SD = 2.4) and preferred therapist gender (M = 5.7, SD = 3.0) had the lowest mean ratings. Paired sample t-tests were conducted to test whether the differences in ratings were significant for the various attributes, and the Holm-Bonferroni method was applied to control for type 1 errors. All differences were significant, expect for self-disclosure and training institution (*p* = 0.10) as well as area of expertise and years of experience (*p* = 0.17).

### Fear of COVID-19 scores and rating of psychologists depending on area of expertise

To test whether higher levels of fear of COVID-19 resulted in a greater preference for psychologists specialized in anxiety disorders and fear who also mention treating fear of COVID-19, a Factorial Analysis of Covariance (ANCOVA) was conducted with diagnostic status as a categorical covariate and ratings as the dependent variable. Respondents were divided into three groups based on their fear of COVID-19 scores; low fear (FCV-19S score from 7 to 16, *n* = 56), moderate fear (FCV-19S score from 17 to 25, *n* = 87) and high fear (FCV-19S score from 26 to 35, *n* = 44). The results indicated that there was a significant difference between the groups (*F*_(2,183)_ = 3.18, *p* = 0.044). Further, the respondents in the high fear group (M = 6.9, SD = 1.8) were significantly more likely than respondents in the low fear group (M = 6.0, SD = 1.9) to give a higher rating to psychologists who mention treating fear of COVID-19 (MD = 0.92, SE = 0.365, 95% CI = [0.198, 1.64], *p* = 0.013). The difference in mean rating between the high fear (M = 6.9, SD = 1.8) and medium fear group (M = 6.4, SD = 1.7) was not statistically significant (MD = 0.47, SE = 0.333, 95% CI = [-0.193, 1.12], *p* = 0.165). Diagnostic status was not a significant covariate (*F*_(1,183)_ = 0.47, *p* = 0.828).

A second analysis was conducted using ANOVA to compare the high fear of COVID-19 group’s rating of psychologists depending on area of expertise. The results indicated that there was a significant difference between the groups (*F*_(2,129)_ = 3.83, *p* = 0.024). Tukey’s HSD showed that respondents with a high fear of COVID-19 rated psychologists specialized in anxiety disorders and fear (also fear of COVID-19) (M = 7.0, SD = 1.7) significantly higher than generalist psychologists (M = 5.6, SD = 2.8) (MD = 14, SE = 5.03, 95% CI = [1.99, 25.88], *p* = 0.018). The difference between psychologists specialized in anxiety disorders and fear (also fear of COVID-19) and psychologists specialized in anxiety disorders and fear (M = 6.3, SD = 2.4) was non-significant (MD = 6.4, SE = 5.03, 95% CI = [-5.56, 18.33] *p* = 0.416). The difference between specialized in anxiety disorders and fear and generalist psychologists was also non-significant (MD = 7.5, SE = 5.03, 95% CI = [-19.49, 4.40], *p* = 0.295). In conclusion, the results indicate that clients who are more fearful of COVID-19 show a higher preference for psychologists who are specialized in anxiety disorders and fear if they explicitly mention treating fear of COVID-19 as well.

### Diagnostic status and fear of COVID-19 score

To test whether UK residents with a preexisting mental health diagnosis showed higher levels of fear than non-diagnosed residents, an independent samples t-test was conducted between diagnosed (*n* = 43) and non-diagnosed respondents (*n* = 144). Diagnosed respondents had a higher mean score of COVID-19 fear (M = 21.7) than non-diagnosed respondents (M = 19.7), however, the difference was not statistically significant (t_(185)_ = 1.68, MD = -2.06, 95% CI = [-4.49, 0.365], *p* = 0.309).

### Ratings of self-disclosure depending on diagnostic status and fear of COVID-19 score

An independent sample t-test was done to compare diagnosed and non-diagnosed respondents’ ratings of self-disclosing psychologists. No significant difference was found between diagnosed (M = 6.02) and non-diagnosed respondents (M = 6.11) (t_(185)_ = 0.273, MD = 0.088, 95% CI = [-0.548, 0.724], *p* = 0.785). To test for differences in ratings between the low, medium, and high fear of COVID-19 group, we conducted an ANOVA which showed significant group differences (*F*_(2,184)_ = 4,12, *p* = 0.018). Tukey’s HSD revealed that the high fear group (M = 6.65, SD = 1.87) rated self-disclosing psychologists significantly higher than the low fear group (M = 5.60, SD = 1.97) (MD = 1.05, SE = 0.37, 95% CI = [0.184,1.92], *p* = 0.013). The difference between the high fear and medium fear group (M = 6.11, SD = 1.69) was not significant (MD = 0.54, SE = 0.34, 95% CI = [-0.26, 1.34], *p* = 0.247). The medium fear group also did not rate self-disclosure significantly higher than the low fear group (MD = 0.51, SE = 0.312, 95% CI = [-0.23, 1.25], *p* = 0.233).

## Discussion

The present study sought to investigate UK residents’ preferences for specialization and self-disclosure in psychologists’ online self-presentation during the early months of the COVID-19 pandemic. Our sample was collected during mid-April 2020 and showed higher mean levels of fear of COVID-19 compared to an earlier pre-lockdown study in the UK (*n* = 344) in late March 2020 [37]. Other studies done in Italy, New Zealand, France, Brazil, Taiwan and Pakistan between March and May 2020 also show comparatively lower fear of COVID-19 mean scores [38]. Though our sample is not representative, prevalence of diagnosed mental health disorders was 23% in the present study. This is a bit lower than the 31 and 32% prevalence estimates for anxiety and depression found in a meta-analysis of studies done in the UK between March 23 and May 13 2020, but higher than the 2014 Adult Psychiatric Morbidity Survey report which indicated a 17–18% prevalence of common mental health disorders [39, 40].

Earlier studies have suggested that individuals with preexisting mental health conditions, particularly anxiety, are more vulnerable and exposed to exacerbation of mental distress related to COVID-19 [3, 41]. In the present study, we did not find diagnosed UK residents to exhibit more fear of COVID-19 compared to non-diagnosed UK residents. However, this could be because we did not distinguish between preexisting mental conditions and that we didn’t measure multiple domains of mental distress [41].

In support of the first hypothesis, our findings indicated that UK residents with a high fear of COVID-19 prefer psychologists specializing in anxiety disorders and fear (including fear of COVID-19). Earlier studies have found support for student populations’ preference for clinical psychologists over educational psychologists, psychiatrists, clinical social workers, and counsellors, but few empirical studies have looked at preferences for de facto specialization [13, 16, 17]. Still, several scholars have suggested specialization is beneficial for both clients and therapists [18–21].

In the present study, specialization was the second most important attribute in UK residents’ rating of psychologist profiles—indicating that listing de facto specialization might ease client decision-making. This is an important finding considering that distressed individuals researching treatment options often experience reduced cognitive function that might delay seeking help [42]. During the pandemic, stress on cognitive function was further exacerbated by social isolation during lockdown and COVID-19 information overload through news and social media [43, 44].

Our second hypothesis stated that UK residents with a high fear of COVID-19 or a mental health diagnosis would show preference for psychologists disclosing a mild history of mental illness. This was only partially supported by our findings. Diagnostic status was not differentially associated with preferences in self-disclosure. In fact, our confidence interval suggested that non-diagnosed residents would be more likely to give self-disclosing psychologists a higher rating upon repeated sampling. In contrast, UK residents with a high fear of COVID-19 did show preference for self-disclosing psychologists as compared to residents with a low fear of COVID-19.

Very little research has looked at preference for self-disclosure based on diagnosis, but self-disclosure in general has received support from a handful of studies which have investigated self-disclosure in therapy sessions [25–27, 45]. These studies have found positive associations between self-disclosure and ratings of therapists, expected success of therapy and a better working relationship overall [25–27]. In the current study, however, the relative importance score and utility estimates indicate that self-disclosure was not a very important preference for UK residents in general. This could reflect the categorical operationalization of the construct and the severity of the mental illness disclosed. Thus, overall, our findings suggest personal branding attributes might facilitate decision-making for some clients—with specialization being more important than self-disclosure.

Findings for other psychologist attributes in the present study indicate that years of experience and gender were the most influential preferences in ratings of psychologists (along with area of expertise). Self-disclosure, training institution and facial trustworthiness were of less importance. In terms of utility levels, it was found that UK residents prefer psychologists with 15 ± 1 years of experience, who are specialized, female, disclose a past history of mild mental illness, graduated from a highly ranked training institution and have more trustworthy faces. The findings for training institution are similar to other conjoint studies on preferences for mental health professionals [13, 16, 17]. However, gender had a comparatively high importance score in the present study.

This study is, to the authors’ knowledge, the first conjoint analysis using pictures of hypothetical psychologists in measuring preferences in choice of psychologist. In addition to adding realism to the design, facial trustworthiness was chosen, because it is one of the most important facial dimensions in social cognition [46]. Facial trustworthiness is also correlated with facial attractiveness and may even share a similar neural substrate [46, 47]. This correlation is relevant as earlier studies have found physically attractive therapists are perceived as more intelligent, friendly, trustworthy, reliable, sincere, and skilled [48, 49]. The current study found a small preference for more trustworthy faces, yet this attribute had a low relative importance.

The importance of trustworthiness, more broadly, was also measured in the rating of individual attributes where respondents gave trustworthiness the highest rating followed by area of expertise, years of experience, self-disclosure, training institution, and preferred therapist gender. Interestingly, gender was rated as much less important when attributes were rated in isolation. This could reflect the use of images in the conjoint profiles. Respondents’ high rating of trustworthiness is supported by an abundance of theoretical literature in psychotherapy which argue that trust is an essential aspect of the therapeutic alliance [50]. Few studies, however, have directly measured clients’ level of trust towards psychotherapists [50]. It should also be mentioned that although several preferences have been found to be important in this study, earlier research suggests that clients’ preferences might be malleable, thus, it is probable that client preferences can be influenced through different forms of online self-presentation [48, 49, 51–53].

Several limitations of the present study should be noted. Firstly, due to budget constraints, our sample size and statistical power was small, but our study makes a novel contribution to the research field, both in terms of design, timing of data collection and findings. In addition, our results are relevant for future meta-analyses on client preferences. Secondly, the external validity of our study is limited. By design, conjoint studies usually entail fixing some attributes that are not of primary interest but help increase realism. In the present study, we fixed facial expressions and ethnicity. Images with smiling, open-mouth faces were chosen as we assume psychologists generally seek to exude warmth and approachability towards prospective clients.

Ethnicity was restricted to white faces which is somewhat representative as 88.2% of clinical psychologists in the UK are white according to a report by the BPS from 2015 [54]. Nevertheless, as white respondents constituted 80.2% of our sample, we recognize this as a possible confounder, but would also like to emphasize that ethnicity was not a primary research objective in present study. Preference for facial similarity between respondents and the profile images might also have influenced our findings, but as we asked respondents to rate facial trustworthiness in each profile, we believe to have controlled for this factor to some extent as individuals tend to view self-resembling faces as more trustworthy [55–57].

Hypothetical bias has also been raised as a concern regarding conjoint studies, but earlier research has indicated that this is possibly less prevalent in choice experiments in the health domain [58]. A similar concern is the experimental demand effect in which participants respond to confirm the researchers’ hypothesis [59]. However, conjoint analysis might actually help reduce this bias, as multiple variables are included in the design, making it less evident which hypothesis to favor and to what degree [59]. Respondent fatigue is also frequently raised as an issue in conjoint studies, but several studies have pointed to this problem as being overstated [60, 61], in addition, the current study entails comparatively fewer profiles compared to similar studies [11–13].

Based on the limitations in the present study, future research could help shed more light on preference for specialization in clinical and ethnic populations with specific mental disorders. More research on levels and types of self-disclosure depending on client type would also give a better sense of preferences for this attribute. Lastly, conjoint studies using different constellations of attributes and levels (e.g., length and content of profile texts, use of videos etc.) would further help clarify how important preferences for specialization and self-disclosure are.

## Conclusions

The present study highlights the importance of how psychologists are presented online within the context of the COVID-19 pandemic. More specifically, personal branding—in the form of emphasizing de facto specialization—might facilitate clients’ decision-making process when considering therapy. Self-disclosure might also be of benefit to specific help-seeking clients (e.g., residents with a high fear of COVID-19), but in the present study, self-disclosure was not a very important attribute in general whereas specialization was highly important along with years of experience. Though our study was conducted during the beginning of a global pandemic, we believe our findings are relevant for post-pandemic times as well. Psychologists presented in online directories or on private practice websites should help clients exercise their freedom to choose whom they seek help from; thus, public and private mental health care providers should consider what attributes are presented and how they either facilitate or hamper clients’ decision-making process.

## Data Availability

The dataset analysed in the current study is not publicly available because no explicit consent has been given for open sharing, however, the data can be made available for individual researchers on reasonable request to the corresponding author.
